# Machine learning the Hohenberg-Kohn map for molecular excited states

**DOI:** 10.1038/s41467-022-34436-w

**Published:** 2022-11-17

**Authors:** Yuanming Bai, Leslie Vogt-Maranto, Mark E. Tuckerman, William J. Glover

**Affiliations:** 1 NYU Shanghai, 1555 Century Avenue, 200122 Shanghai, China; 2 NYU-ECNU Center for Computational Chemistry at NYU Shanghai, 3663 Zhongshan Road North, 200062 Shanghai, China; 3 Department of Chemistry, New York University, New York, NY 10003 USA; 4 Simons Center for Computational Physical Chemistry at New York University, New York, NY 10003 USA; 5 Courant Institute of Mathematical Science, New York University, New York, NY 10012 USA

**Keywords:** Density functional theory, Method development, Chemical physics, Excited states

## Abstract

The Hohenberg-Kohn theorem of density-functional theory establishes the existence of a bijection between the ground-state electron density and the external potential of a many-body system. This guarantees a one-to-one map from the electron density to all observables of interest including electronic excited-state energies. Time-Dependent Density-Functional Theory (TDDFT) provides one framework to resolve this map; however, the approximations inherent in practical TDDFT calculations, together with their computational expense, motivate finding a cheaper, more direct map for electronic excitations. Here, we show that determining density and energy functionals via machine learning allows the equations of TDDFT to be bypassed. The framework we introduce is used to perform the first excited-state molecular dynamics simulations with a machine-learned functional on malonaldehyde and correctly capture the kinetics of its excited-state intramolecular proton transfer, allowing insight into how mechanical constraints can be used to control the proton transfer reaction in this molecule. This development opens the door to using machine-learned functionals for highly efficient excited-state dynamics simulations.

## Abstract (web summary)

Density functional theory provides a formal map from the electron density to all observables of interest of a many-body system; however, maps for electronic excited states are unknown. Here, the authors demonstrate a data-driven machine learning approach for constructing multistate functionals.

## Introduction

Electronic excitations underlie numerous biological and physical processes of interest, including photosynthesis^1,2^, DNA damage^3,4^, photodynamic therapy^5^, photopharmacology^6,7^, and solar energy conversion^8,9^. Excited-state dynamics simulations are a powerful tool to uncover the mechanism of photoresponse in these systems, particularly when combined with ab initio electronic-structure calculations, since the important molecular motions do not need to be known a priori but are revealed by the dynamical trajectories^10,11^. Given its reasonable accuracy within current approximations, linear-response time-dependent density-functional theory (LR-TDDFT)^12,13^ has become a work-horse method in the field; however, its computational expense (formally scaling with system size as *N* ^3^) and the need to solve the electronic-structure problem at every simulation timestep limits the system size and timescale amenable to study. Thus, a cheaper approach to electronic excitations and excited-state dynamics is highly desirable but poses a significant challenge.

One might question whether the expense of LR-TDDFT is needed in the first place. For the ground state (GS), the first Hohenberg–Kohn (HK1) Theorem^14^, which provides the foundation of DFT, establishes the existence of a bijection from the ground-state electron density to the external potential of a many-body system. As a consequence, there exists a formal map from the ground-state electron density not only to the ground-state energy but also to every property of the system. The map from density to ground-state energy is encoded by an unknown universal density functional; however, practical approaches make use of the Kohn-Sham (KS) theory^15^, which expresses the functional via a fictitious non-interacting system that shares the density of the true system, allowing an exact treatment of kinetic energy and Coulomb contributions to the functional and approximations to the remaining small exchange and correlation terms. While KS theory provides a practical scheme for solving simultaneously for the ground-state density and energy of a system, functionals that map from the ground-state density to excited-state energies are currently unknown, although, their formal existence is established by the HK1 theorem. As an alternative, LR-TDDFT provides an indirect means to the excited-state energies from knowledge of the ground-state density and its response to an external driving field^12,13^. Given the expense of LR-TDDFT, a direct map from density to excited-state energies would be preferable.

Theoretical support for a direct route to excited-state energies comes from a generalization of the HK1 theorem to an excited-state density-energy bijection. While a universally general HK1 theorem for excited states with arbitrary external potentials has been disproved^16^, a special excited-state HK1 theorem for Coulombic external potentials (i.e., molecular systems) has been argued to exist^17^ and has motivated recent orbital-optimized DFT approaches to electronic excited states^18,19^.

Alongside theoretical developments in excited-state DFT, there has been significant recent progress in using data-driven approaches to machine-learn ground-state density functionals^20–28^. Related to the current work, there has also been progress in developing machine-learning models to directly predict electronic excited-state energies, gradients, and non-adiabatic couplings given a particular molecular geometry, and the SchNarc approach by Westermayr, Marquetand et al. has been demonstrated to predict non-adiabatic dynamics in agreement with ab initio simulations using a reasonable amount of training data^29–35^. In the current work, we explore the proposition that a multistate density functional can be machine learned via an excited-state HK map. This new development establishes a framework that is potentially more powerful than directly learning excited-state energies, since densities and density functionals can yield any desired property.

In order to construct an ML excited-state density functional, we work in the framework introduced in ref. 22, which uses a data-driven ML model with a physically motivated representation of the molecule. The fundamental object in this approach is a potential-to-density map, *n*[*v*](**r**), which is machine-learned by computing the external potential and corresponding density at a desired level of theory for a range of nuclear geometries of the system and then inputting these functions as training data. For ground-state densities, this map is known as the machine-learned Hohenberg–Kohn (ML-HK) map; once learned, it can be used to compute any related quantities, including ground-state energies, and other observables at the same level of theory^22,23^. It is worth noting that the level of theory is not restricted to DFT but can also be performed, for example, using coupled-cluster theory^23^. In this work, we generalize the potential-to-density map to excited states in a multistate Hohenberg–Kohn framework (ML-MSHK), allowing us to learn excited-state densities and energies simultaneously with comparable accuracy to the ground state.

In order to test our ML-MSHK model, we consider the excited-state proton transfer (ESPT) reaction in malonaldehyde (MA), a small organic molecule exhibiting a non-trivial internal reaction that is sensitive to electronic excitation. ESPT is at the heart of photoacidity and is a key step in the photocycle of many photoactive proteins, such as green fluorescent protein (GFP)^36,37^. Intramolecular proton transfer, as in MA, serves as a useful model to study ESPT, since the reaction rate is not limited by the diffusion of the proton donor and acceptor together, and can therefore be probed by ultrafast spectroscopy^38,39^.

**Fig. 1:**
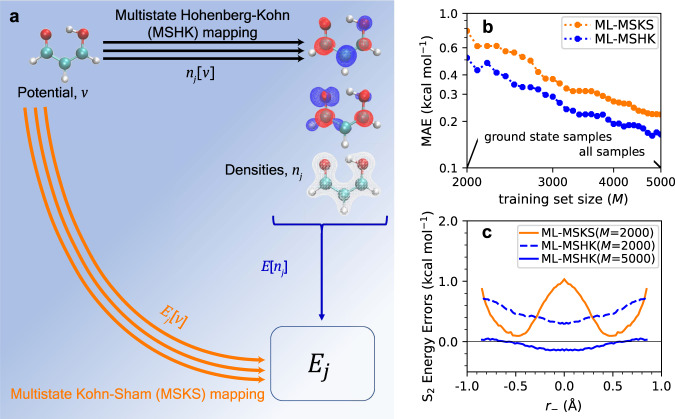
Overview and performance of the multistate machine-learning (ML) approach. **a** DFT maps used in this paper. The orange arrows represent a direct ML-MSKS map from the external potential, *v*, to total electronic energies, *E*_*j*_, for each state *j*. The black arrows represent ML-MSHK maps between *v* and electron densities of each state, *n*_*j*_. The blue arrow represents a single multistate energy functional that maps a density to its energy. The structure of the malonaldehyde (MA) molecule studied here is shown in the top left of this panel. **b** Learning curves (on a logarithmic scale), for MA’s S_2_ energy predictions from the ML-MSHK model (blue curve) using the lowest three densities (*n*_0_, *n*_1_, *n*_2_) and ML-MSKS model (orange curve). Training sets are formed starting from 2000 geometries sampled from S_0_ dynamics and sequentially adding geometries extracted from ab initio molecular dynamics (AIMD) trajectories on the S_2_ state in the manner described in Supplementary Note 1. **c** Errors of energy prediction against a TD-PBE0 benchmark along a minimum energy pathway for proton transfer of MA in the S_2_ state predicted with ML-MSKS and ML-MSHK maps using training sets of different sizes. For each fixed value of proton-transfer coordinate, *r*_−_ (see text for definition), geometries were optimized on S_2_ at the TD-PBE0/aug-cc-pvdz level, subject to a constraint of planarity.

The structural simplicity of MA makes it an especially appealing model of ESPT. In particular, one conformation of malonaldehyde is a ring structure with an internal hydrogen bond that allows for a proton-transfer reaction between the two oxygen atoms (see molecular graphic in Fig. 1a). Excitation of this molecule from the S_0_ ground state to the S_2_ singlet excited state (ππ^*^) leads to a substantial reduction of the proton-transfer barrier from several kcal/mol in the ground state to an essentially barrierless reaction in the excited state. However, the presence of a lower-lying singlet nπ^*^ state in MA significantly complicates its ESPT reaction, and ultrafast non-adiabatic transitions from S_2_ to S_1_ are believed to compete with the ESPT reaction^40–42^. The barrier to proton transfer on S_1_ is even higher than the ground-state^40,43^, and furthermore, a three-state intersection is predicted to be energetically accessible^41^, meaning the electronic excitation can be efficiently quenched to the ground state. As a result, the ESPT reaction in MA is largely hindered by competing processes and has yet to be directly observed experimentally^42^.

One approach to suppressing the competing processes in MA and, thereby, controlling the ESPT reaction is to raise the nπ^*^ state energy via functional group modifications. For example, the nπ^*^ and ππ^*^ energetic ordering is reversed in methyl salicylate (MS)^44^, which shares the chelate ring of MA yet exhibits efficient ESPT, as the non-adiabatic transitions to the nπ^*^ state are removed^38,45,46^. In this work, using excited-state dynamics with our ML-MSHK method, we seek to explore whether mechanical restrictions applied to MA can instead be used to promote the ESPT reaction. Recognizing that the S_2_/S_1_ transition in MA is brought about by torsional motions^41^, we propose that a restraint of planarity on the molecule will allow the ESPT reaction to proceed unhindered. This restraint can be viewed as biomimetic of a similar mechanism in GFP whose protein environment enforces planarity of the 4-(p-hydroxybenzylidene)imidazolidin-5-one chromophore, preventing nonadiabatic transitions and enhancing its fluorescence quantum yield^47^.

## Results

### Theory

There is a growing recognition of the importance of using physics-based principles in choosing descriptors and models for machine-learning molecular properties^48,49^. The blessing, and perhaps curse, of ML models is their great flexibility to predict almost any quantity given sufficient data. This can lead to a loss of physical insight, and concerns of overfitting must always be addressed. By using physical principles, fundamental constraints are automatically introduced into the model that mitigate these problems. In the context of learning electronic structure, a very natural physical framework relies on the connections between the electron density, *n*(**r**), external potential, *v*(**r**), and energy, *E*, of a many-body quantum system. These connections are formalized by the HK1 theorem, which proves the existence of a bijection from the ground-state electron density to the external potential of a many-body system: *n*_0_(**r**) ↔ *v*(**r**)^14^. The electron density, which gives the probability for finding an electron at a certain location in space, is a particularly convenient quantity to work with since, unlike the many-body wavefunction, it is a three-dimensional scalar field regardless of the number of electrons and is, therefore, straightforward to represent numerically. For molecular systems, the external potential is simply the Coulombic scalar potential arising from the nuclear charges: $v({{{{{{{\bf{r}}}}}}}})=-\mathop{\sum }\nolimits_{a}^{N}{Z}_{a}/|{{{{{{{\bf{r}}}}}}}}-{{{{{{{{\bf{r}}}}}}}}}_{a}|$. Since the external potential uniquely defines the molecular electronic Hamiltonian (for a given spin state and number of electrons), the electronic energy must also be uniquely determined from knowledge of the external potential or, per HK1, the density^14^. These maps from external potential to density and energy are realized as functionals, e.g., *n*[*v*](**r**), which inspired the framework for a previous approach that machine-learned density functionals for ground-state energy predictions (ML-HK)^22^. Part of the success of the ML-HK approach can be attributed to the uniqueness of the external potential and density as molecular descriptors, a property not shared by some other choices^50^.

We now demonstrate how these ideas can be extended to electronic excited states. As mentioned in the introduction, an excited-state extension of HK1 has been argued to exist for Coulombic external potentials^17^, meaning that the map from external potential to each state’s density is encoded as a functional, *n*_*j*_[*v*](**r**), where *j* = 0, 1, 2, . . . . labels each excited state. Furthermore, a property of the (unknown) exact universal functional is that each extremal density of the energy functional corresponds to an electronic eigenstate with the functional returning the exact (excited-state) eigenvalue, *E*_*j*_ = *E*[*n*_*j*_]^51^. This motivates a machine-learning approach that leverages an excited-state Hohenberg–Kohn mapping.

### Learning excited states with ML-MSHK

The first step of our ML model is to learn multiple electronic state densities. We start by expanding the densities in an orthonormal basis set, *ϕ*_*l*_(**r**), as ${n}_{j}^{{{{{{{{\rm{ML}}}}}}}}}[v]=\mathop{\sum }\nolimits_{l=1}^{L}{u}_{j}^{(l)}[v]{\phi }_{l}({{{{{{{\bf{r}}}}}}}})$, and we learn the set of basis coefficients $\{{u}_{j}^{(l)}\}$ by training on a set of input potentials corresponding to different geometries of a system. We allow for the learning of ground- and excited-state densities with a unique map to each state *j*. Various ML models, including artificial neural networks and kernel methods, have been used to learn total energies from electronic-structure calculations^22,23,30,52–59^. However, learning excited-state HK maps is unique to our approach, and we follow the ground-state ML-HK functional’s successful use of the kernel ridge regression (KRR) method^60^. In principle, the functionals could instead be learned with neural networks, but a comparison of different methods is beyond the scope of this manuscript.

With a set of learned densities, a second KRR model is used to learn ground- or excited-state energies from a set of input training densities. We consider two types of energy functionals, depending on the nature of the input used. The first relies on the proven existence of a single functional that maps ground or excited-state densities to their respective energy eigenvalues^51^. We thus learn a single map from density to energy and use multiple states in the training of this map:

1 $${E}^{{{{{{{{\rm{ML-MSHK}}}}}}}}}[{n}_{j}]=\mathop{\sum }\limits_{i=1}^{M}\mathop{\sum}\limits_{k}{\alpha }_{i,k}K({{{{{{{{\bf{u}}}}}}}}}_{i,j}[v],{{{{{{{{\bf{u}}}}}}}}}_{i,k}[{v}_{i}]),$$

where *K*(**u**_*i*,*j*_[*v*], **u**_*i*,*k*_[*v*_*i*_]) is the kernel, *k* runs over the states used in the training of the model, and {*α*_*i*,*k*_} are the coefficients learned in the energy functional model. Since energy predictions from this model rely on training with multiple electron densities for a given molecular geometry, we call the combination of the two types of maps the multistate Hohenberg–Kohn approach (ML-MSHK). Learning Eq. (1) comes at the cost of retaining multiple densities for each training example, increasing storage needs; however, the advantage is the resulting energy functional is not state specific. We note that while this work focuses on the planar S_2_ state, we also train on densities from the S_1_ excited state to demonstrate that including additional densities does not degrade the performance of the ML-MSHK model.

The second energy functional we consider is of the external potential itself, without using the density as an intermediate descriptor. Following the naming of a similar approach for ground-state energies^22^, we call this the multistate Kohn-Sham map (ML-MSKS):

2 $${E}_{j}^{{{{{{{{\rm{ML-MSKS}}}}}}}}}[v]=\mathop{\sum }\limits_{i=1}^{M}{\gamma }_{i,j}K\left[{v}_{i},v\right],$$

where {*γ*_*i*,*j*_} are the coefficients learned in the ML-MSKS energy functional model. Such functionals must also exist since the external potential uniquely defines the molecular electronic Hamiltonian and, therefore, also its eigenstates^14^. Eq. (2) has the advantage that it uses only external potentials rather than densities, reducing computational storage requirements compared to Eq. (1). However, a disadvantage is that the energy maps in this model are state specific, which we expect will introduce errors near electronic crossings^30^.

In all cases, models are trained against ab initio densities and energies following the procedure described in section “Methods”. A schematic of the different maps used in this study is shown in Fig. 1a.

### Excited-state energy predictions

In order to test the performance of the excited-state machine-learned density functionals, we start by considering learning curves for training S_2_ excited-state energies of MA, shown in Fig. 1b, in which the lowest three densities (*n*_0_, *n*_1_, *n*_2_) were used for the ML-MSHK model. The smallest training set considered contains 2000 molecular conformations of MA generated from a ground-state AIMD trajectory, propagated as described in section “Training and test set generation”. A training set similar to this was previously found to yield a ML-HK potential that accurately described the proton-transfer reaction on the ground state^22^. From Fig. 1b, however, we see somewhat large out-of-sample mean absolute errors (MAEs) of 0.5 and 0.8 kcal/mol for the ML-MSHK and ML-MSKS models, respectively, with this training set. Furthermore, for the ML-MSKS model, the error is seen to vary strongly with geometry, as revealed by the orange curve in Fig. 1c, which shows energy prediction errors along a minimal-energy pathway for the ESPT reaction as a function of the proton-transfer coordinate, *r*_−_ = *r*_HO1_ − *r*_HO2_, where *r*_HO*i*_ is the distance of the proton from oxygen atom *i*. Using the same 2000 training geometries, the ML-MSHK error (dashed blue curve) is more uniform than the ML-MSKS predictions, demonstrating the advantage of the former.

In order to use the ML-MSHK model for excited-state dynamics, we must further reduce its prediction errors; however, we found essentially no improvement upon adding more samples from the ground-state trajectory. As we will see below, the remaining source of error in models trained only with ground-state samples is that the S_2_-initiated ESPT involves nuclear responses also in modes orthogonal to the direct proton transfer mode, and these are not adequately sampled by the ground-state trajectory. Thus, we extended the training set by including geometries extracted from 30 S_2_ excited-state AIMD trajectories, in a manner described in Supplementary Note 1, yielding the learning curves shown in Fig. 1b.

After including geometries extracted from excited-state trajectories in the training (ground and excited-state samples), both ML models perform significantly better than the ML model constructed using only ground-state samples in the training. Interestingly, the ML-MSKS map (Fig. 1b, orange curve) always performs worse than the MSHK map (blue curve). Similar behavior was noted in the ground-state ML-HK study of ref. 22. The rest of this work pertains to the ML-HK models since learning the densities as an intermediate step yields the models that outperform the direct potential-to-energy approach, and the inclusion of density learning has other benefits such as the capability of density-based delta-learning to add corrections from high-level wavefunction theory^23^. Encouragingly, the out-of-sample error for ML-MSHK converges below 0.2 kcal/mol once the sample size reaches *M* = 5000. This error is comparable to that found in the previous ground-state study of MA^22^, suggesting that ML-MSHK will be suitable for molecular dynamics simulations (confirmed below). Having found convergence at *M* = 5000, our final training set generation follows the protocol described in section “Training and test set generation”. For the adiabatic excited-state dynamics considered in this work, it is possible to train state-specific ML-HK models to return the S_2_ energies using only ground-state or S_2_ densities as input (see Supplementary Fig. 1). While we do not include any non-adiabatic transitions in this study, we note that the state-specific models are worse than ML-MSHK in the vicinity of electronic crossings (see Supplementary Note 5). Given the excellent performance of the ML-MSHK model and its ability to capture multiple electronic states simultaneously, we focus exclusively on this model for the remainder of this work.

### Excited-state density predictions

**Fig. 2:**
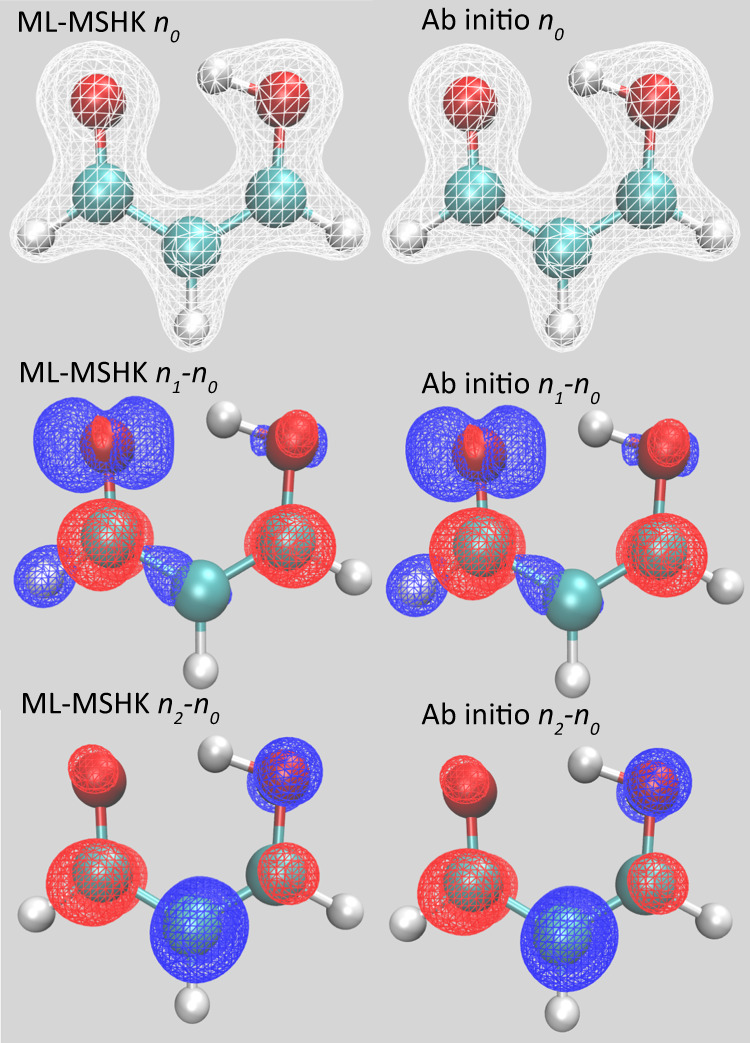
Ground and excited-state electron densities for the *C*_*S*_ ground-state minimum energy structure of MA. 1st row: S_0_ ground-state densities. 2nd row: density differences between S_1_ and S_0_. 3rd row: density differences between S_2_ and S_0_. An isosurface of 0.1 *e*/Bohr^3^ was used for plotting densities and density differences. Left column: ML predictions, right column: ab initio TD-PBE0 predictions. Each density is represented by an isosurface plot. For density differences, red means an accumulation of electronic charge in the excited state and blue means a depletion.

Equally important to energy predictions is the question of how well the model reproduces electron densities. This is explored in Fig. 2, which shows electron densities for the *C*_*S*_-symmetric ground-state optimized geometry of MA (similar results for the proton transfer transition state are shown in Supplementary Fig. 4). In order to highlight the changes in bonding structure, excited-state densities are displayed as density differences with respect to the ground state. For all states considered, there is no discernible difference between the ML-MSHK predicted densities (left) and ab initio TD-PBE0 densities (right), even though the ground-state optimized structure was not included in the training set. Indeed, the out-of-sample integrated MAE in the total electron densities is very small (0.012 *e*).

Knowledge of the density differences also provides insight into the nature of the electronic excitations. From Fig. 2, S_1_ clearly corresponds to an nπ^*^ transition with a depletion of density from the proton-accepting oxygen’s *p*_*x*_ orbital and an accumulation on the backbone π^*^ orbital. S_2_, on the other hand, is a ππ^*^ transition, as identified by a depletion of density on the central conjugated carbon’s *p*_*z*_ orbital.

### Excited-state MD using ML potentials

Having demonstrated that our ML-MSHK model provides a quantitative prediction of excited-state electron densities and total energies within our test set of geometries, we next consider its ability to generate a functional for use in excited-state MD. We thus initiated non-equilibrium MD trajectories of MA on its S_2_ state following vertical excitation from the ground state. 1000 ML-MSHK and 50 AIMD trajectories were run and compared. It should be noted that the computational effort associated with propagating dynamics on the ML-MSHK surface is negligible compared to AIMD dynamics. Thus, for the cost of 50 excited-state AIMD trajectories used to generate the training and test sets, we gain the ability to perform excited-state dynamics with 1000 ML-MSHK trajectories using the resulting machine-learned functional to converge the excited-state non-equilibrium averaged properties.

**Fig. 3:**
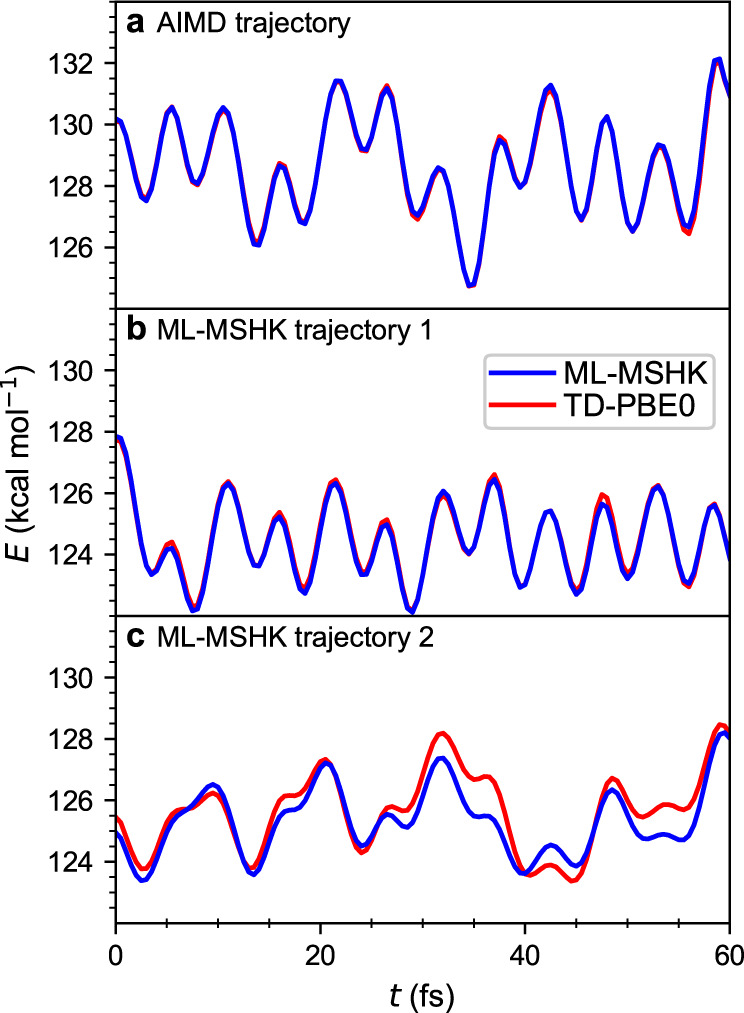
Predictions of electronic energies along trajectories. ML-MSHK predictions (blue curves) and TD-PBE0 reference values (red curves) are shown along three different representative trajectories taken from: **a** an ab initio molecular dynamics (AIMD) trajectory and **b** an ML-generated excited-state trajectory, both initiated from geometries in the training set, and **c** an ML-generated excited-state trajectory initiated from a geometry out of the training set.

We consider three metrics to assess the quality of the machine-learned functional. The first concerns how well ML-MSHK is able to interpolate the energy between geometries explicitly included in the training. This is accomplished by evaluating ML-MSHK energies along an AIMD excited-state trajectory from which some geometries were included in the training. The result is shown in Fig. 3a, where we see almost perfect agreement between ML-MSHK and the AIMD TD-PBE0 energies. Note that only 32 geometries out of 481 from this trajectory were used in the training set of the ML-MSHK model, highlighting the fidelity to which ML-MSHK is able to interpolate the functional between training points. Second, we assess how well ML-MSHK propagates the dynamics starting from a geometry included in the training set, shown in panel b, where again we see almost perfect agreement with AIMD energies evaluated on the ML trajectory snapshots. Finally, we check how well the machine-learned functional does on arbitrary geometries by evaluating ab initio TD-PBE0 energies along an ML-MSHK trajectory where no geometry was included in the training set, shown in panel c. Here, slightly larger deviations between ML-MSHK and TD-PBE0 energies are seen; however, the difference is within the expected MAE bounds from the learning curve and does not grow with time, showing that ML-MSHK faithfully reproduces the excited-state energy functional, at least in the configurational space sampled during non-equilibrium dynamics. The error could be reduced by further increasing the training set; however, we found this unnecessary since this small error does not influence the dynamical predictions as we show below.

### ESPT in planar MA

Having seen that ML-MSHK provides an accurate description of the S_2_ excited-state functional of MA, we next consider the predictions of excited-state dynamics of the ESPT reaction using this functional. To that end, we compute the following non-equilibrium response function:

3 $$S(t)=\overline{\left({r}_{-}(0){r}_{-}(t)\right)/\left|{r}_{-}(0){r}_{-}(t)\right|},$$

where *t* = 0 corresponds to the instance of vertical excitation from an equilibrium configuration on the S_0_ state to the S_2_ state, and the overbar indicates a non-equilibrium average over initial conditions. *S*(*t*) thus measures the memory of which reaction basin the proton is in (reactant or product). A value of *S* = 1 means the proton is in the reactant basin, a value of *S* = − 1 means the proton is in the product basin, and a value of *S* = 0 means memory has been lost. Since MA has a symmetric proton donor and acceptor, *S* = 0 indicates the reaction is complete.

**Fig. 4:**
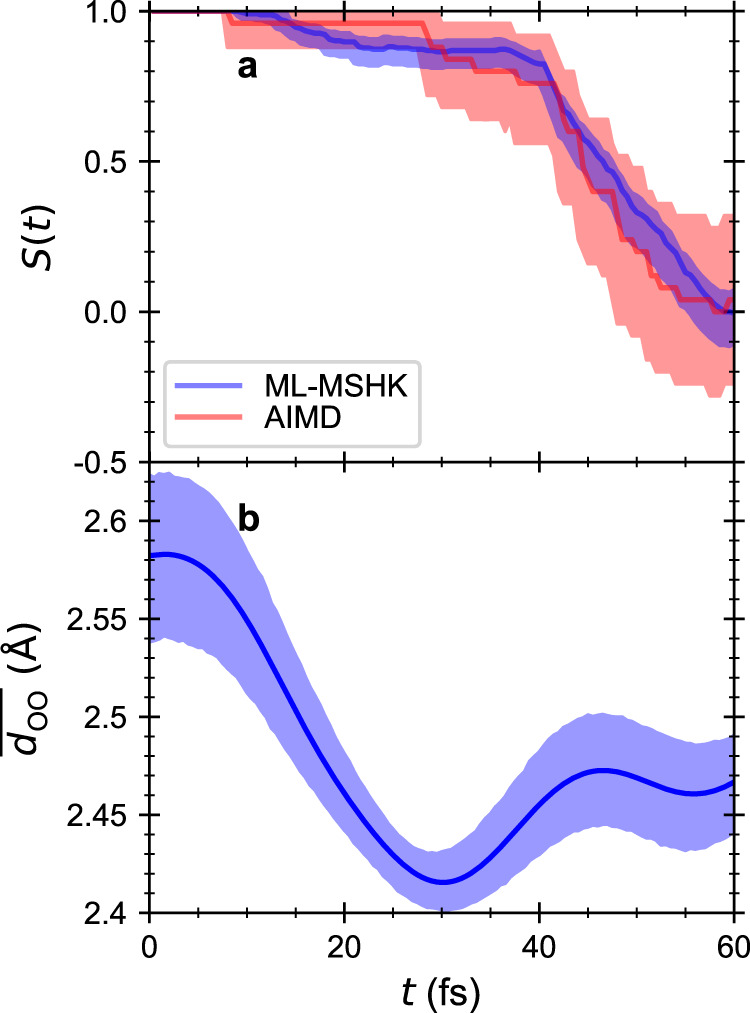
The non-equilibrium response of the excited-state proton transfer reaction and its relationship to the time varying oxygen–oxygen distance of MA. **a** Non-equilibrium response function of the proton’s location (reactant or product basin according to Eq. (3)), *S*(*t*), that reflects the progress of the excited-state proton transfer reaction. The solid curves are the expectation values of *S*(*t*) and the shaded area shows the 95% confidence intervals estimated from bootstrap analysis using 50 trajectories for ab initio molecular dynamics (AIMD) (red) and 1000 trajectories (blue) for ML-MSHK. The converged *S*(*t*) of the ML-MSHK model shows good agreement with the result from AIMD simulations. **b** The average oxygen–oxygen distance is shown as a function of time following photoexcitation.

Figure 4a shows the ESPT reaction’s non-equilibrium response function, *S*(*t*), computed for 50 AIMD trajectories (red) and 1,000 ML-MSHK trajectories (blue). Shaded areas represent the 95% confidence intervals computed with the bootstrap method^61^, as implemented in SciPy v. 1.7.1^62^. The results show that the AIMD and ML-MSHK agree within statistical certainty, highlighting the predictive power of ML-MSHK. Furthermore, an interesting feature of *S*(*t*) becomes clear in the ML-MSHK results: apart from a ~10% fraction of trajectories that undergo almost immediate proton transfer, there is a waiting period of ~40 fs before the remainder of ESPT reaction proceeds, which is then rapidly completed by 60 fs. This behavior is hinted at in the AIMD results but becomes much more apparent in the ML-MSHK predictions due to averaging over many more trajectories.

The observed 40-fs waiting period arises from important responses of the heavy atoms following excitation to S_2_. This can be seen for example in the average oxygen–oxygen distance, $\overline{{d}_{{{{{{{{\rm{OO}}}}}}}}}}$, plotted in Fig. 4b, which exhibits a decrease from ~2.57 Å to ~2.42 Å in the first 30 fs following excitation. This timescale is comparable to the waiting period of the ESPT reaction, suggesting that the oxygen–oxygen motion gates the reaction, which does not proceed rapidly until a distance of *d*_OO_ ≤ 2.45 Å is reached.

**Fig. 5:**
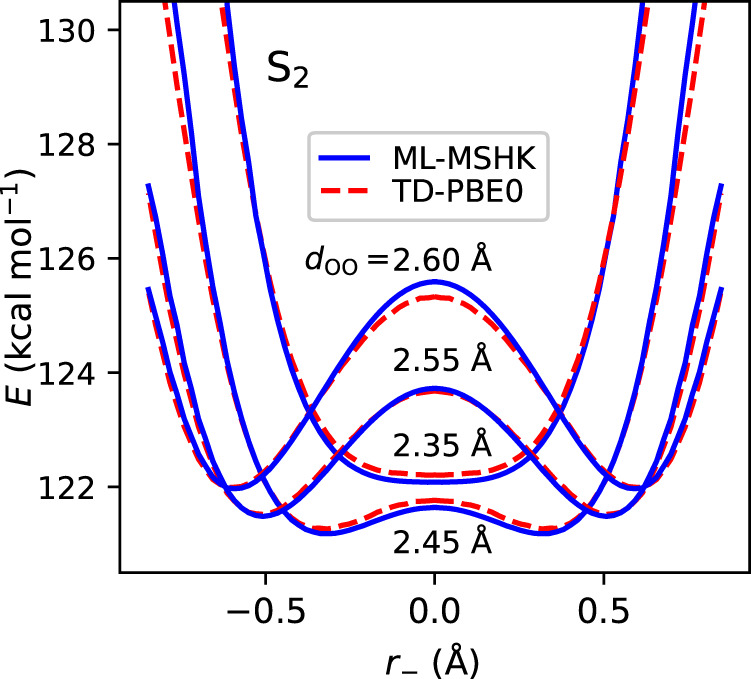
S_2_ potential energy surfaces of MA along the H-transfer coordinate, *r*_−_, for a series of fixed oxygen–oxygen distances, *d*_*oo*_. All coordinates except *r*_−_ and *d*_*oo*_ are optimized at the TD-PBE0/aug-cc-pvdz level for S_2_, subject to a constraint of planarity, following the procedure described in Supplementary Note 3. We see excellent agreement between ab initio results (dashed red curves) and ML-MSHK predictions (solid blue curves).

The response of *d*_OO_ following excitation to S_2_ can be understood from changes in the electron density shown in Fig. 2. The reduction in *π*-bonding character lessens the rigidity of the conjugated system, allowing the chelating ring to contract and the oxygen atoms to become closer. Bringing together the proton donor and acceptor oxygen atoms then reduces the barrier to ESPT, thus explaining the gating mechanism. This is explored explicitly in Fig. 5, which plots the potential energy profile of the minimum energy pathway of ESPT on S_2_ for a series of fixed *d*_OO_ distances. Encouragingly, quantitative agreement between ML-MSHK (blue curves) and TD-PBE0 (dashed red curves) is seen, despite none of the minimum energy pathway geometries being included explicitly in the training set. For the initial average *d*_OO_ distance (~2.57 Å), a proton-transfer barrier of >2 kcal/mol is seen, explaining why only a small fraction of excited trajectories undergo ESPT in the first 20 fs. The S_2_ potential is downhill for motions that reduce *d*_OO_ to a value of 2.45 Å (following the minimum of the potential energy profile as a function of *d*_OO_), which also brings about a reduction of the barrier to proton transfer, having a value of 0.3 kcal/mol at *d*_OO_ = 2.45 Å. The barrier disappears completely for *d*_OO_ = 2.35 Å; however, this oxygen–oxygen distance is disfavored due to an overall increase in the potential, explaining why the initial reduction in *d*_OO_ is seen to reverse at *t* = 30 fs in Fig. 4b.

## Discussion

In this paper, we developed a multistate Hohenberg–Kohn machine-learning framework for the accurate prediction of excited-state densities and energies. We demonstrated that excited-state energies, which are expressible as a single functional of multiple state densities, can be learned as accurately as the ground-state energy, even for the moderately sized molecule malonaldehyde, which exhibits a non-trivial excited-state proton transfer reaction. The resulting machine-learned functional is faithful to the underlying excited-state AIMD trajectories on which the model was based, allowing accurate non-equilibrium excited-state molecular dynamics trajectories to be performed with a 10-fold computational saving compared to AIMD. Furthermore, our study yielded new insight into the excited-state proton transfer reaction in malonaldehyde. This was aided by the low-cost of ML-MSHK energy predictions that allowed us to converge the non-equilibrium dynamics with 1,000 excited-state trajectories, revealing the 40-fs waiting period following photoexcitation, after which ESPT promptly completed.

The observed gating mechanism of proton transfer in planar MA by heavy atom motion has been seen previously in other intramolecular ESPT reactions which are not precluded by ultrafast non-adiabatic transitions. In particular, the lack of isotope effect for ESPT in methyl salicylate was explained by a delocalization of the reaction coordinate to modes other than the donor OH stretch^38^. Later studies in related ESPT molecules found the reaction path was dominated by anharmonic low-frequency backbone modes that modulated the donor-acceptor distance^63–65^. Thus, by enforcing planarity in MA, we have suppressed nonadiabatic transitions that compete with ESPT and find the mechanism of proton transfer is indeed multidimensional in nature and follows the concensus picture that has emerged for intramolecular chelate ring structures. That we correctly captured the multidimensional nature of ESPT from a limited training of excited-state trajectories is a testament to the power of our ML-MSHK method.

This work can be extended in a number of directions. As an example, we removed the restraint of planarity on malonaldehyde (see Supplementary Note 5). Models using additional non-planar training geometries show that the multistate density functional outperforms the state-specific functionals near S_2_/S_1_ state crossings, which is vital for running future non-adiabatic MD trajectories. In addition, the LR-TDDFT training data could be replaced with high-level wavefunction-based electronic structure for input to train the ML-MSHK density and energy maps. A similar approach was recently shown to be successful for the ground-state ML-HK method to learn coupled-cluster energies^23^. Based on the promising results presented in this work, we are optimistic that ML-MSHK will find practical use in excited-state non-adiabatic simulations with the costly electronic-structure steps done only once to build the training set.

## Methods

### Machine-learning model

In order to predict the excited-state energies of a molecule using electron densities via the ML-MSHK map introduced in section “Learning excited states with ML-MSHK”, we start by using a basis expansion of the densities:

4 $${n}_{j}^{{{{{{{{\rm{ML}}}}}}}}}[v]({{{{{{{\bf{r}}}}}}}})=\mathop{\sum }\limits_{l=1}^{L}{u}_{j}^{(l)}[v]{\phi }_{l}({{{{{{{\bf{r}}}}}}}}),$$

where *j* indexes an electronic state (*j* = 0 is the ground state, *j* = 1 is the first excited state, etc). Following the ground-state ML-HK method^22^, we choose a Fourier basis. In Eq. (4), *l* indexes a basis function, of which there are *L*. In this work 50 functions are used in each dimension (125,000 = 50 × 50 × 50 in total). The coefficients of the basis set expansion are learned using KRR. In particular, ${u}_{j}^{(l)}$ are represented as a kernel expansion of the form

5 $${u}_{j}^{(l)}[v]=\mathop{\sum }\limits_{i=1}^{M}{\beta }_{i,j}^{(l)}\kappa [{v}_{i},v].$$

Here, ${\beta }_{i,j}^{(l)}$ parameterizes the kernel model, and the kernel functional *κ* has a Gaussian form

6 $$\kappa [{v}_{i},v]=\exp \left[-\frac{1}{{\sigma }^{2}}\int\ {{{{{{{\rm{d}}}}}}}}{{{{{{{\bf{r}}}}}}}}{\left(v({{{{{{{\bf{r}}}}}}}})-{v}_{i}({{{{{{{\bf{r}}}}}}}})\right)}^{2}\right],$$

where *σ* is a kernel width hyperparameter. The external potentials, *v*_*i*_(**r**), *i* = 1, . . . , *M*, are unique to each of the *M* training geometries, and are paired with *M* training densities, *n*_*i*,*j*_(**r**), for each state *j*. Like the density, we use a basis representation of the external potential; however, care must be taken to avoid the Coulomb singularities in *v*(**r**). We therefore use a Gaussian representation of the potential:

7 $$v({{{{{{{\bf{r}}}}}}}})=\mathop{\sum }\limits_{a}^{N}{Z}_{a}{e}^{-|{{{{{{{{\bf{R}}}}}}}}}_{a}-{{{{{{{\bf{r}}}}}}}}{|}^{2}/2{\sigma }_{{{{{{{{\rm{pot}}}}}}}}}^{2}},$$

where **R**_*a*_ is the position of the *a*th nucleus, *Z*_*a*_ is its corresponding nuclear charge, and *σ*_pot_ is a width parameter^22,66^. The potential from Eq. (7) is then formed on a 3D grid surrounding the molecule and stored as a vector, **v**_*i*_, for each sample *i* to be used in Eq. (6). 60 × 50 × 30 grid points were used with a spacing of 0.2 Å, commensurate with the shape of the molecule. We used previously optimized values of *σ*_pot_ = 0.2 Å and a grid spacing for MA from ref. 22.

As a final step, we train a multistate total energy functional:

8 $${E}^{{{{{{{{\rm{ML-MSHK}}}}}}}}}[{n}_{j}]=\mathop{\sum }\limits_{i=1}^{M}\mathop{\sum}\limits_{k}{\alpha }_{i,k}\kappa [{{{{{{{{\bf{u}}}}}}}}}_{i,k},{{{{{{{{\bf{u}}}}}}}}}_{j}],$$

where *k* sums over the states of the densities used in the training and *κ* is another Gaussian kernel.

### Reference ab initio electronic structure

Reference calculations on MA’s excited states were performed at the LR-TDDFT level using the PBE0^67,68^ approximate exchange and correlation functional. This level of theory, which we abbreviate as TD-PBE0, was chosen as a compromise between accuracy and efficiency in this first demonstration of our ML-MSHK method. In particular, PBE0 provides qualitatively correct ground-state thermochemical properties of MA, yielding proton transfer barrier height of 2.0 kcal/mol compared to the predicted barrier from high-level theory of 4.1 kcal/mol^69,70^. In addition, TD-PBE0 provides reasonable spectroscopic quantities for MA, with a S_0_-S_2_ vertical excitation of 5.3 eV compared to the experimental value of 4.7 eV^71^. Electronic structure calculations were performed in CPMD v. 4.3.0^72^ using a plane-wave basis with a kinetic energy cutoff of 90 Rydberg. Core electrons were replaced with Troullier-Martins norm-conserving pseudopotentials^73^.

### AIMD excited-state molecular dynamics

Ab initio excited-state Born-Oppenheimer MD simulations of a gas-phase malonaldehyde molecule were performed in CPMD using the same TD-PBE0 level of theory discussed above. 50 independent non-equilibrium trajectories were initiated on the S_2_ state following vertical excitations spaced every 100 fs from an AIMD ground-state trajectory sampled at 300 K taken from ref. 22. Excited-state dynamics were propagated in the microcanonical ensemble with a 0.25 fs timestep for 120 fs, which takes 5027 min for one simulation with all cores (24) of dual Intel Xeon E5 2650 v4 (2.2 GHz) CPUs. Planarity was maintained with the following added restraining potential: ${V}^{rest.}=\mathop{\sum }\nolimits_{a}^{N}\frac{1}{2}{k}_{a}{({z}_{a})}^{2}$, where *k*_*a*_ is the strength of each restraint and is 9 kcal/(mol bohr^2^) for hydrogen and 40 kcal/(mol bohr^2^) for heavy atoms. *z*_*a*_ is the z-coordinate of each atom. The planarity restraints were chosen to be stiffer for the heavy atoms, since backbone torsions have been identified as bringing about the S_2_/S_1_ electronic crossing in MA^41^.

### Training and test set generation

It has been shown that exploiting molecular point group symmetries helps to increase the effective dataset size significantly without performing additional quantum chemical calculations^23^. We thus generated training sets for malonaldehyde that included reflection about the mirror symmetry plane perpendicular to the molecular axis, ${\sigma }_{v{\prime} }$, of an idealized planar C_2*v*_ structure, since this symmetry operation ensures the equivalence of the two oxygen atoms as proton donors/acceptors. To achieve a symmetrization of the training sets while avoiding unnecessary duplication of samples, we combined symmeterization with a clustering of molecular geometries. For example, the training set for MD runs was formed by starting with 2000 structures taken from a previous ground-state AIMD trajectory^22^ and 14,400 structures taken every timestep from 30 independent ab initio excited-state trajectories described in section “AIMD excited-state molecular dynamics”. Next, all geometries were aligned to a reference geometry representing the planar proton-transfer transition state with C_2*v*_ symmetry (see Supplementary Note 4). Then, following alignment, any molecular structures with a negative proton-transfer coordinate, *r*_−_, were reflected in the ${\sigma }_{v{\prime} }$ plane, i.e., to ensure *r*_−_ > 0. The training set was then clustered with *K*-Means^74^ to make a set of 2500 samples, using a metric related to the *L*^2^ deviation of the external potential, $\sqrt{\int\ {{{{{{{\rm{d}}}}}}}}{{{{{{{\bf{r}}}}}}}}{\left({v}_{i}({{{{{{{\bf{r}}}}}}}})-{v}_{j}({{{{{{{\bf{r}}}}}}}})\right)}^{2}}$, where *v*_*i*_ represents the potential from Eq. (7). Finally, the training set was doubled in size by applying the reflection operator in the ${\sigma }_{v{\prime} }$ plane to yield the production training set of 5000 geometries. The test set contains 240 aligned snapshots extracted every 10 fs from 20 independent ab initio excited-state trajectories described in section “AIMD excited-state molecular dynamics”.

The electronic-structure calculations for excited-state energies and densities take 40 min with 8 cores of an Intel Xeon E5 2650 v4 (2.2 GHz) CPU for one geometry and the training takes 10 min using the same number of cores with hyperparameters given.

### ML excited-state molecular dynamics

To generate initial conditions to perform excited-state dynamics on the ML S_2_ potential, a ground-state AIMD trajectory was run in CPMD using the PBE exchange and correlation functional^67^ with the same kinetic energy cutoff and pseudopotentials discussed above. This trajectory was run in the canonical ensemble at 300 K using massive Nosé-Hoover chain thermostats with a timestep of 0.5 fs^75^. 1000 independent non-equilibrium trajectories were initiated on the S_2_ state following vertical excitations spaced every 100 fs from the ground-state trajectory. Excited-state dynamics were propagated in the microcanonical ensemble with a 0.25 fs timestep using the atomistic simulation environment v.3.19.0^76^. Atomic forces were evaluated numerically using central differences with a step length of *dx* = 0.001 Å. Dynamics were propagated for 60 fs, which was sufficient to observe the ESPT reaction. The propagation of one 60-fs dynamics simulation takes 20 min with 2 cores of an Intel Xeon E5 2650 v4 (2.2 GHz) CPU.

## Supplementary information

Supplementary Information

## Data Availability

The molecular coordinates, electronic densities and energies for ground and excited states used in this study are available in *Zenodo* [10.5281/zenodo.7064211]. The source data of the figures are provided in the Source Data file. Source data are provided with this paper.

## Source data

Source Data

## Code availability

Codes used in this work are available at 10.5281/zenodo.7064211.
