# Supplementary material for: Machine learning the Hohenberg-Kohn map for molecular excited states
Source: Nat Commun. 2022 Nov 17;13:7044. doi: 10.1038/s41467-022-34436-w (PMC9672065; doi:10.1038/s41467-022-34436-w)
Supplement: Supplementary file 1 — Supplementary Information [file 41467_2022_34436_MOESM1_ESM.pdf]

Supplementary Information for 'Machine learning the Hohenberg-Kohn map for molecular excited states'

Yuanming Bai,[1,2,3] Leslie Vogt-Maranto,[3] Mark E. Tuckerman,[2,3,4,5] and William J. Glover[1,2,3,*]

[1]*NYU Shanghai, 1555 Century Avenue, Shanghai 200122, China*
[2]*NYU-ECNU Center for Computational Chemistry at NYU Shanghai, 3663 Zhongshan Road North, Shanghai 200062, China*
[3]*Department of Chemistry, New York University, New York, New York 10003, USA*
[4]*Simons Center for Computational Physical Chemistry at New York University, New York, NY 10003, USA*
[5]*Courant Institute of Mathematical Science, New York University, New York, NY, 10012, USA*

Supplementary Method 1. Kernel Ridge Regression

As described in the main text, we use Kernel Ridge Regression (KRR) to machine learn excited-state density functionals. The key equations of KRR are briefly reviewed here in terms of abstract training points $(\mathbf{x}_i, \mathbf{y}_i)$, where $\mathbf{x}_1, ...\mathbf{x}_M \in \mathbb{R}^d$ are the features and $\mathbf{Y} = (y_1, ...y_M)^T \in \mathbb{R}^M$ their respective labels. We seek a function that maps from the features to the labels $f : \mathbb{R}^d \to \mathbb{R}$, and this is expressed as a linear expansion of the kernel function, κ:

$$f = \sum_{i=1}^{M} \boldsymbol{\alpha}_i \kappa\left(\mathbf{x}_i, \cdot\right), \tag{1}$$

where $\boldsymbol{\alpha}_i$ are the model coefficients to be found, and the kernel has a Gaussian form:

$$\kappa\left(\mathbf{x}, \mathbf{x}'\right) = \exp\left(-\frac{\|\mathbf{x} - \mathbf{x}'\|^2}{2\sigma^2}\right). \tag{2}$$

Here σ controls the width of the Gaussian kernel, and is optimized as a hyperparameter during the model training.

The model is trained by minimizing the following loss function:

$$\min_f \left\{\sum_{i=1}^{M} \left|\mathbf{y}_i - f\left(\mathbf{x}_i\right)\right|^2 + \lambda \|f\|_{\mathcal{H}}^2\right\} = \min_{\boldsymbol{\alpha}} \left\{\sum_{i=1}^{M} \left|\mathbf{y}_i - f\left(\mathbf{x}_i\right)\right|^2 + \lambda \boldsymbol{\alpha}^\top \mathbf{K} \boldsymbol{\alpha}\right\}, \tag{3}$$

where $\mathbf{K}_{ij} = \kappa\left(\mathbf{x}_i, \mathbf{x}_j\right)$ is the kernel matrix, $\|f\|_{\mathcal{H}}$ is the reproducing kernel Hilbert space norm, and λ is a regularization parameter which, like σ, is optimized as a hyperparameter. The solution of minimizing the loss function (Supplementary Eq. 3) is

$$\boldsymbol{\alpha} = (\mathbf{K} + \lambda \mathbf{I})^{-1} \mathbf{Y}. \tag{4}$$

In the context of the ML-KS map, the training features are discretized Gaussians potentials and the respective labels are their associated excited-state energies. To learn the electron density representations, the training features are the discretized Gaussians potentials and the respective labels are the set of Fourier coefficients for each energy state. In the context of the energy functionals, the training features are the expansion coefficients of the electronic density in a Fourier basis representation and the labels are the associated excited-state energies.

Supplementary Method 2. ML multistate Hohenberg-Kohn map

As described in Section IV A, we use a basis representation of the electronic densities for a particular electronic state, given by

$$n(\mathbf{r}) = \sum_{l=1}^{L} u^{(l)} \phi_l(\mathbf{r}), \tag{5}$$

[*] Email to: william.glover@nyu.edu

where ϕ_l are the Fourier basis functions of which there are L. To train the model, first the basis set coefficients, $\{u^{(l)}\}$, are found by a Fourier transform of the density from a regular real-space grid representation:

$$u^{(l)} = \sum_{m=1}^{L} n(\mathbf{r}_m) e^{-i2\pi \boldsymbol{k}_l \cdot \mathbf{r}_m / \boldsymbol{B}}, \tag{6}$$

where $\{\mathbf{r}_m\}$ are the real-space grid points on which the density is evaluated, $\boldsymbol{k}_l$ is the wavevector of Fourier function l, and $\boldsymbol{B}$ are the real-space grid box lengths. In our implementation, we instead perform sine and cosine transformations (equivalent to a Fourier transform) so that the frequency domain density coefficients are purely real numbers. If L_x is the number of sine and cosine functions of the x coordinate, then the total number of coefficients is $L = L_x \times L_y \times L_z$.

For a particular electronic state, the loss function for machine-learned densities follows similarly to the regular KRR model:

$$e(\boldsymbol{\beta}) = \sum_{i=1}^{M} \left\| n_i - n^{\text{ML}}[v_i] \right\|_{\mathcal{L}_2}^2 + \lambda \|n\|_{\mathcal{H}}^2. \tag{7}$$

By writing the ML model with basis function coefficients and a kernel expansion as in section IV A, $n^{\text{ML}}[v_i] = \sum_{l=1}^{L} \sum_{i=1}^{M} \beta_{i,j}^{(l)} \kappa[v_i, v] \phi_l(\mathbf{r})$, the loss function becomes:

$$e(\boldsymbol{\beta}) = \sum_{i=1}^{M} \left\| n_i - \sum_{l=1}^{L} \sum_{k=1}^{M} \beta_k^{(l)} \kappa[v_i, v_k] \phi_l \right\|_{\mathcal{L}_2} + \lambda \boldsymbol{\beta}^T \mathbf{K} \boldsymbol{\beta}. \tag{8}$$

Here, $\kappa[v_i, v_k]$ is a kernel functional given by Supplementary Eq. 2 with $\|\cdot\|$ representing the function norm of the function $v_i(\mathbf{r}) - v_k(\mathbf{r})$.

Using the orthogonality of the basis functions yields

$$e(\boldsymbol{\beta}) = \sum_{i=1}^{M} \sum_{l=1}^{L} \left| \mathbf{u}_i^{(l)} - \sum_{k=1}^{M} \boldsymbol{\beta}_k^{(l)} \kappa[v_i, v_k] \right|^2 + \sum_{l=1}^{L} \lambda [\boldsymbol{\beta}^{(l)}]^T \mathbf{K} \boldsymbol{\beta}^{(l)}, \tag{9}$$

the solution of which is analogous to that of regular KRR:

$$\boldsymbol{\beta}^{(l)} = (\mathbf{K}_\sigma + \lambda \mathbf{I})^{-1} \mathbf{u}^{(l)}, \quad l = 1, \ldots, L \tag{10}$$

where λ is a global regularization hyperparameter and $\boldsymbol{K}_\sigma$ is the Gaussian kernel with a global kernel width hyperparameter, σ. These hyperparameters were optimized in a grid search (following Ref. 1) with the standard 5-fold cross-validation procedure.[2, 3]

Supplementary Method 3. Clustering the training set

For a molecule with several degrees of freedom, like malonaldehyde, the accuracy of a machine-learned density functional will depend on both the size of the training set and how well the training set samples the important conformational space of the molecule of interest. To generate an efficient training set for planar malonaldehyde, we follow previous work[1] and employ K-means clustering to identify the important and diverse samples that we then include in the training set to cover the relevant conformational space as much as possible with a limited number of conformers.

Assuming we have conformers labelled $i = 1...N$ with parameters $\mathbf{p}_i$, we seek to find M clusters, $\tilde{\mathbf{P}}_j, j = 1...M$, that minimize the following objective function:

$$O = \sum_{j=1}^{M} \sum_{i \in \tilde{\mathbf{P}}_j} \|\tilde{\mathbf{p}}_j - \mathbf{p}_i\|^2, \tag{11}$$

where $i \in \tilde{\mathbf{P}}_j$ if and only if $\tilde{\mathbf{p}}_j$ is the cluster center closest to $\mathbf{p}_i$. In principle, $\mathbf{p}_i$ can be any properties of the conformer. In this work we used the Gaussian representation of the external potential $v(\mathbf{r})$, since this property enters the Gaussian kernel of Supplementary Eq. 9, and exhibits desirable features, such as permutational invariance. The K-means algorithm provides a solution to Supplementary Eq. 11,[4] and we select the M samples closest to the cluster centers.

Supplementary Note 1. Convergence of the model with sample size

To generate a learning curve that reflects the benefits of adding training data from an increasing number of non-equilibrium excited-state trajectories, we used an iterative approach to expand our training set. Starting with the 1000 representative ground state samples which were clustered and aligned from the 2000 ground state samples as described in Section IV D, 480 aligned geometries from a single S_2 trajectory were added to the training set pool. All 1480 geometries were clustered with the K-means algorithm to identify 1050 trajectory snapshots closest to the cluster centers, i.e. the training set size was expanded by 50 samples each iteration. This procedure was iterated 30 times, eventually yielding 2500 samples before symmetrization, and the 31 training sets generated from this procedure were used in Fig. 1 to get the mean absolute error on the test set of S_2 excited state geometries. In this way, information from one new trajectory was added to the model at a time. The learning curve showed that when we had added 30 excited state trajectories, the out-of-sample error was already below 0.2 kcal mol^{-1}. In the final training set, 817 of the original unsymmeterized ground state snapshots survived, with the remainder of the samples comings from the added excited-state trajectories.

Supplementary Note 2. Two additional frameworks for learning excited-state functionals

In addition to the ML-MSHK framework using multiple states in the training described in the main text, we also tested two different frameworks for predicting excited-state energies. Both approaches can only be used to predict the S_2 excited state energies. We call them ML-ESHK[n_2] and ML-ESHK[n_0].

The first model, ML-ESHK[n_2], leverages the excited-state HK theorem, but uses only a single excited-state density, n_2, in training the functional. As a result, ML-ESHK[n_2] is a state-specific version of ML-MSHK and shows a similar performance away from electronic crossings. The second model, ML-ESHK[n_0], reflects a map from ground-state densities, n_0, to excited-state energies, and can be viewed as an excited-state version of the original ML-HK approach.

The learning curves for ML-ESHK[n_2] and ML-ESHK[n_0], are shown in Supplementary Figure 1, using the same training set as in Fig. 1b. The performance of the two models is comparable to ML-MSHK. However, both ML-ESHK[n_2] and ML-ESHK[n_0] share the disadvantage of being state specific (similar to the ML-MSKS model) so we focus on ML-MSHK in the main text.

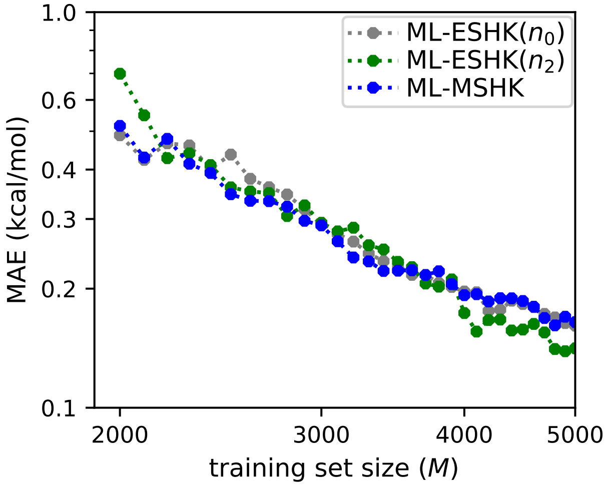

Supplementary Figure 1: Learning curves for planar malonaldehyde's S_2 energy predictions for ML-MSHK (blue), ML-ESHK[n_0] (grey), and ML-ESHK[n_2] (green) on a logarithmic scale. Here, ML-ESHK[n_0] is a map from ground-state densities to S_2 excited-state energies and ML-ESHK[n_2] is a map from S_2 excited-state densities to S_2 excited-state energies. The training sets are the same as in Fig. 1b.

Supplementary Note 3. Malonaldehyde excited-state proton transfer minimum energy pathway

The potential energy profile in Fig. 5 was generated with the CIOpt software package.[5] The geometries corresponding to each point along the curves in this figure were optimized in internal coordinates for a series of fixed proton-transfer coordinates (r_-) and oxygen-oxygen distances (d_{OO}), subject to a constraint of planarity. The electronic structure of the S_2 state was found using TD-PBE0[6, 7] with the aug-cc-pvdz basis set[8] as implemented in TeraChem[9, 10]. With the minimal energy pathways found, TD-PBE0 energies were evaluated along the pathways using CPMD[11], in order to make a direct connection to our ML-MSHK model, which was trained on CPMD energies.

Supplementary Note 4. C_{2v} geometry for alignment

As discussed in section IV D, each sample is aligned to a C_{2v} geometry representing an idealized planar proton-transfer transition state of malonaldehyde. The detailed coordinates of this C_{2v} geometry are shown here.

```
9

H  -2.1630  -0.5470   0.0000
H   0.0000  -1.7840   0.0000
H   2.1630  -0.5470   0.0000
H   0.0000   1.6650   0.0000
C  -1.2150   0.0000   0.0000
C   0.0000  -0.7010   0.0000
C   1.2150   0.0000   0.0000
O  -1.3130   1.2870   0.0000
O   1.3130   1.2870   0.0000
```

Supplementary Note 5. Learning excited-state functionals for non-planar Malonaldehyde

In the main text, we considered planar malonaldehyde, which maintains a large S_2-S_1 energy gap for the duration of the excited-state proton transfer reaction. In this section, we explore the ability of machine-learned excited-state functionals to predict densities and energies relevant to the S_2 excited-state dynamics of MA in the absence of a planar restraint. Previous theoretical studies predict out-of-plane motions on the S_2 state that lead to electronic crossings with lower states.[12] This molecule therefore serves as a useful test of the ability of machine-learned excited-state functionals to describe electronic near-degeneracies. We consider two models: the ML-MSHK model mentioned in the main text and the ML-ESHK model described in Supplementary Note 2. Here the ML-ESHK model uses separate maps to predict S_1 and S_2 densities and energies, i.e. ML-ESHK[n_1] and ML-ESHK[n_2], while the ML-MSHK model simultaneously predicts S_1 and S_2 energies. Because the S_0/S_1 energy gap is larger than 88 kcal/mol along all the ab initio trajectories with TD-PBE0 electronic structure, *i.e.* S_0/S_1 and S_0/S_2 crossings are not observed, we consider only S_1 and S_2 predictions. Not including S_0 densities reduces the storage requirements of the training set.

To generate training and test sets for non-planar MA, we performed 100 additional independent AIMD trajectories on the S_2 state (i.e. adiabatic dynamics) without any restraint of planarity. Initial conditions were taken following vertical excitations spaced every 100 fs from the same AIMD ground-state trajectory used in the main text. Dynamics was propagated for 60 fs using CPMD.[11] Otherwise, all other aspects of the simulations were identical to those described in Section IV C of the main text.

Next, a series of increasingly larger grand training sets were generated by augmenting 5,000 samples from the planar MA training set described in the main text with structures taken from every timestep of the first 20 to 90 non-planar excited-state AIMD trajectories. Each trajectory contributed 240 structures, so we considered grand training sets of between 9,800 and 26,600 samples. 2,400 samples from the remaining ten excited-state AIMD trajectories were used as a test set.

To reduce the size of the training set, we used the dataset reduction protocol of Smith et al.[13] Briefly, starting from 5% of structures randomly selected from the grand training set, an excited-state functional was trained. The energy predictions for the remaining training samples in the grand training set were then evaluated. 2% of samples that had absolute errors in energy (averaged over both S_1 and S_2 in the case of ML-MSHK) of greater than a threshold of 0.3 kcal/mol were randomly selected and added to the training set and the model retrained. Energy predictions for the remaining samples were re-evaluated and another 2% of samples that had energy errors over the threshold were randomly selected and added to the training set. The process was repeated iteratively until fewer than 5% of

the remaining samples had errors above the threshold, at which point, the remaining samples with errors above the threshold were added to the training set.

To further reduce the storage requirements of the training set, compared to the grid used for planar MA, we pruned the number of real-space grid points on which the external potential was evaluated (for use in eq. 6) to $48 \times 40 \times 24$ with a spacing of 0.25 Å. We verified this grid was sufficiently large to span the entire molecule with a buffer of at least 2 Å from any atom to the edge of the grid, even for non-planar geometries. We also confirmed that the increase in grid spacing did not affect the out-of-sample test error.

To improve the learning of densities and assign the appropriate energies, we found it necessary to incorporate a simple state-tracking algorithm when labelling the ab initio states as S_1 or S_2. At each excited-state AIMD timestep, we compared the excited-state densities, expanded in a basis of 27,000 ($30 \times 30 \times 30$) Fourier functions, to the previous timestep's $(t - \delta t)$ densities by computing the inner product,

$$S_{j,k}(t) = \sum_{l}^{L} u_j^{(l)}(t) u_k^{(l)}(t - \delta t), \tag{12}$$

where l runs over the Fourier basis coefficients, and j and k run over the state indices (S_1 and S_2 in our case). If $S_{1,2}(t) + S_{2,1}(t) > S_{1,1}(t) + S_{2,2}(t)$, the adiabatic state labels at timestep t were swapped compared to their ordering at the previous timestep. This tracking accounted for the possibility that the ordering of the electronic states switch as a trajectory traverses an electronic state crossing.

With the sample reduction and state-tracking protocols described above, we generated learning curves for the ML-MSHK and ML-ESHK models by varying the grand training set size from 20 to 90 trajectories, i.e. 9,800 to 26,600 total samples, and evaluating out-of-sample errors on the test set, with the results shown in Supplementary Figure 2. Considering first the ML-ESHK predictions (green curves), the sample reduction protocol is seen to significantly compress the training set; however, the out-of-sample error is much larger than the threshold error. Furthermore, the errors in ML-ESHK appear to saturate and do not improve below 1.5 kcal/mol. The ML-MSHK learning curve, on the other hand, shows that a larger number of samples survive the reduction procedure compared to ML-ESHK; however, the errors now decrease monotonically with increasing size of the grand training set. In particular, the S_1 and S_2 energy predictions attain chemical accuracy compared to the reference ab initio electronic structure (MAEs of 0.90 and 0.83 kcal/mol respectively) at the largest grand training set size (26,600 initial samples and 9,000 samples after reduction).

To confirm that ML-MSHK provides a consistently robust excited-state functional and to understand the lower errors in ML-MSHK compared to ML-ESHK, we plot the predicted excited-state energies along an AIMD trajectory from the test set near S_2/S_1 electronic crossings between $t = 30$ fs to $t = 60$ fs, shown in Supplementary Figure 3. The largest training set following data reduction was used for each model.

ML-MSHK predictions (blue curves) are seen to almost perfectly reproduce the AIMD energies (red curves) for the entire trajectory. On the other hand, the ML-ESHK model (green curves) displays deviations between the predicted and AIMD energies that exceed 2 kcal/mol. In particular, the ML-MSHK prediction error is seen to be noticeably larger after first passing through a crossing region of the electronic states at $t = 42$ fs. To quantify this, we calculated the energy differences before and after the first state crossing, defined as when the S_1/S_2 gap first drops below 10 kcal/mol. After averaging over the test set, we found that the mean absolute error of ML-MSHK is 0.2 kcal/mol before the first state crossing and 1.5 kcal/mol after it. For ML-ESHK, the errors are 0.5 kcal/mol before and 2.4 kcal/mol after. The larger errors for ML-ESHK can be understood as arising from the rapidly changing nature of the electronic density near state crossings, such that the ML-ESHK energy functional, which is trained only on a single state at a time, has insufficient training data to accurately predict the state energy near and following a state crossing. On the other hand, since the ML-MSHK functional is trained simultaneously on both electronic states, it is better able to capture the correct functional dependence on electronic densities in the crossing regions. This clearly demonstrates the benefit of the ML-MSHK model over ML-ESHK.

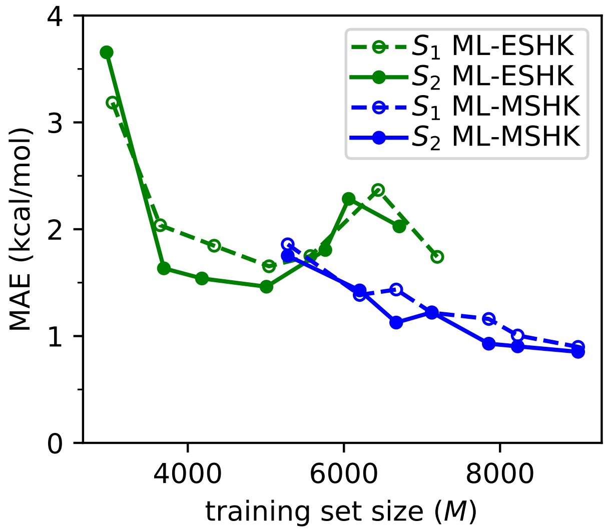

Supplementary Figure 2: Out-of-sample errors for malonaldehyde's energy predictions without planar restraints from two different models (ML-MSHK and ML-ESHK). Grand training sets are formed starting from 5,000 geometries used in the planar training in Section IV D and adding an increasing number of geometries extracted from non-planar AIMD trajectories on the S_2 state. The training set size, M, is the number of samples after applying a sample reduction procedure to each grand training set. The sample reduction was applied separately to the ML-MSHK and ML-ESHK models, resulting in different training set sizes for the two type of models. See the text in Supplementary Note 5. for details.

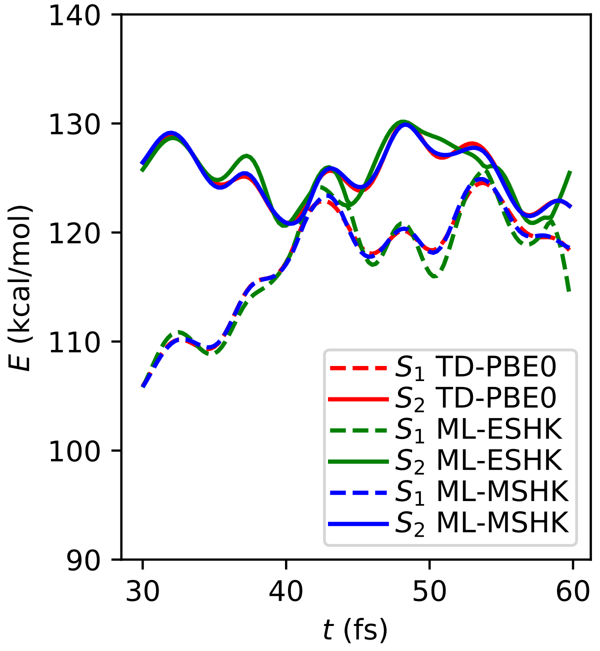

Supplementary Figure 3: Predicted excited-state energies along a non-planar AIMD trajectory in the test set. S_1 and S_2 energies are shown as dashed and solid curves respectively. The ab initio reference (TD-PBE0) is shown in red, ML-MSHK predictions are shown in blue and ML-ESHK predictions in green.

Additional figure

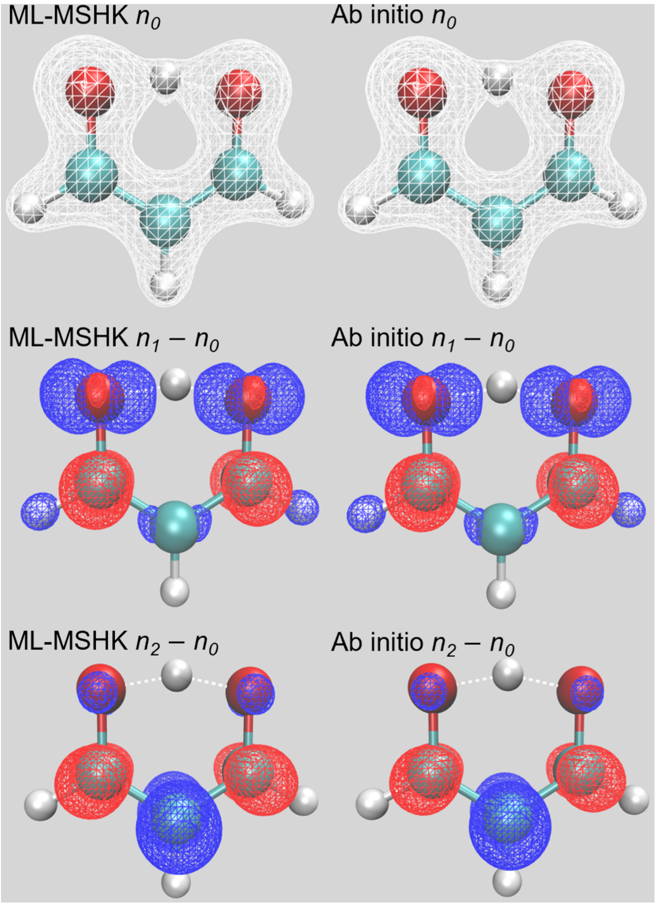

Supplementary Figure 4: Ground and excited-state electronic densities for the C_{2v} halfway proton transfer structure of MA. 1st row: S_0 ground-state densities. 2nd row: density differences between S_1 and S_0. 3rd row: density differences between S_2 and S_0. An isosurface of 0.1 e/Bohr^3 was used for plotting densities and density differences. Left column: ML predictions, right column: ab initio TD-PBE0 predictions. Each density is represented by an isosurface plot. For density differences, red means an accumulation of electronic charge in the excited state and blue means a depletion.

Supplementary References

[1] F. Brockherde, L. Vogt, L. Li, M. E. Tuckerman, K. Burke, and K.-R. Müller, Nat. Commun. **8**, 872 (2017).
[2] K.-R. Muller, S. Mika, G. Ratsch, K. Tsuda, and B. Scholkopf, IEEE Transactions on Neural Networks **12**, 181 (2001).
[3] K. Hansen, G. Montavon, F. Biegler, S. Fazli, M. Rupp, M. Scheffler, O. A. von Lilienfeld, A. Tkatchenko, and K.-R. Müller, J. Chem. Theory Comput. **9**, 3404 (2013).
[4] H. Steinhaus, Bull. Acad. Polon. Sci **1**, 801 (1956).
[5] B. G. Levine, J. D. Coe, and T. J. Martínez, J. Phys. Chem. B **112**, 405 (2008).
[6] J. P. Perdew, M. Ernzerhof, and K. Burke, J. Chem. Phys. **105**, 9982 (1996).
[7] C. Adamo and V. Barone, J. Chem. Phys. **110**, 6158 (1999).
[8] R. A. Kendall, T. H. Dunning, and R. J. Harrison, J. Chem. Phys. **96**, 6796 (1992).
[9] I. S. Ufimtsev and T. J. Martínez, J. Chem. Theory Comput. **4**, 222 (2008).
[10] I. S. Ufimtsev and T. J. Martinez, J. Chem. Theory Comput. **5**, 1004 (2009).
[11] J. Hutter and M. Iannuzzi, Z. Kristallogr. **220**, 549 (2005).
[12] J. D. Coe and T. J. Martínez, J. Phys. Chem. A **110**, 618 (2006).
[13] J. S. Smith, B. Nebgen, N. Lubbers, O. Isayev, and A. E. Roitberg, J. Chem. Phys. **148**, 241733 (2018).
